# The Sandias—Past and Present

**Published:** 1882-05

**Authors:** 


### THE SANDIAS—PAST AND PRESENT.

The Sandia Mountains are dotted with ruins of ancient smelters of a kind that are not in use anywhere at the present day. The native New Mexican or Spanish people do not appear to have acquired the mode of erecting them from the Indians. Even the Pueblo of to-day seems to be ignorant of the manner of their construction. They were cheap and simple, but they answered the purpose admirably. But in no instance has any indication of the existence of a stamp-mill been found in the Sandias. This would show that ancient mining was not restricted to the working of free gold, and that the extraction of gold from smelting ore was understood. But it is undeniable that discoveries in the modes of obtaining gold from combinations of ores have recently been made that could never have been known to the ancients, and this is proven by the fact that much of the slag now found near these old smelters contains gold in considerable quantities. Yet the records, histories, and traditions agree that gold was obtained in fabulous amounts. With modern appliances and new and cheap methods, the yield should now be much greater. Gold ore, in whatever shape, can now be cheaply treated and not an atom be lost. When one takes into consideration the great bulk of gold taken out of the Sandias by the Indians in their crude way, he must be convinced of the wonderful richness of these mountains. There is every reason to believe that the Sandias will eventually be regarded as the greatest of all New Mexican mining districts.—*Bernadillo News.*

